# Human placenta-derived amniotic epithelial cells as a new therapeutic hope for COVID-19-associated acute respiratory distress syndrome (ARDS) and systemic inflammation

**DOI:** 10.1186/s13287-022-02794-3

**Published:** 2022-03-25

**Authors:** Amirhesam Babajani, Kasra Moeinabadi-Bidgoli, Farnaz Niknejad, Hamidreza Rismanchi, Sepehr Shafiee, Siavash Shariatzadeh, Elham Jamshidi, Mohammad Hadi Farjoo, Hassan Niknejad

**Affiliations:** Department of Pharmacology, School of Medicine, Shahid Beheshti University of Medical Sciences, Tehran, Iran

**Keywords:** ARDS, COVID-19, Amniotic membrane, Epithelial stem cells, Immunomodulation, Regenerative medicine, SARS-CoV-2, Exosome

## Abstract

Severe acute respiratory syndrome coronavirus 2 (SARS-CoV-2), which causes coronavirus disease 2019 (COVID-19), has become in the spotlight regarding the serious early and late complications, including acute respiratory distress syndrome (ARDS), systemic inflammation, multi-organ failure and death. Although many preventive and therapeutic approaches have been suggested for ameliorating complications of COVID-19, emerging new resistant viral variants has called the efficacy of current therapeutic approaches into question. Besides, recent reports on the late and chronic complications of COVID-19, including organ fibrosis, emphasize a need for a multi-aspect therapeutic method that could control various COVID-19 consequences. Human amniotic epithelial cells (hAECs), a group of placenta-derived amniotic membrane resident stem cells, possess considerable therapeutic features that bring them up as a proposed therapeutic option for COVID-19. These cells display immunomodulatory effects in different organs that could reduce the adverse consequences of immune system hyper-reaction against SARS-CoV-2. Besides, hAECs would participate in alveolar fluid clearance, renin–angiotensin–aldosterone system regulation, and regeneration of damaged organs. hAECs could also prevent thrombotic events, which is a serious complication of COVID-19. This review focuses on the proposed early and late therapeutic mechanisms of hAECs and their exosomes to the injured organs. It also discusses the possible application of preconditioned and genetically modified hAECs as well as their promising role as a drug delivery system in COVID-19. Moreover, the recent advances in the pre-clinical and clinical application of hAECs and their exosomes as an optimistic therapeutic hope in COVID-19 have been reviewed.

**Graphical abstract:**

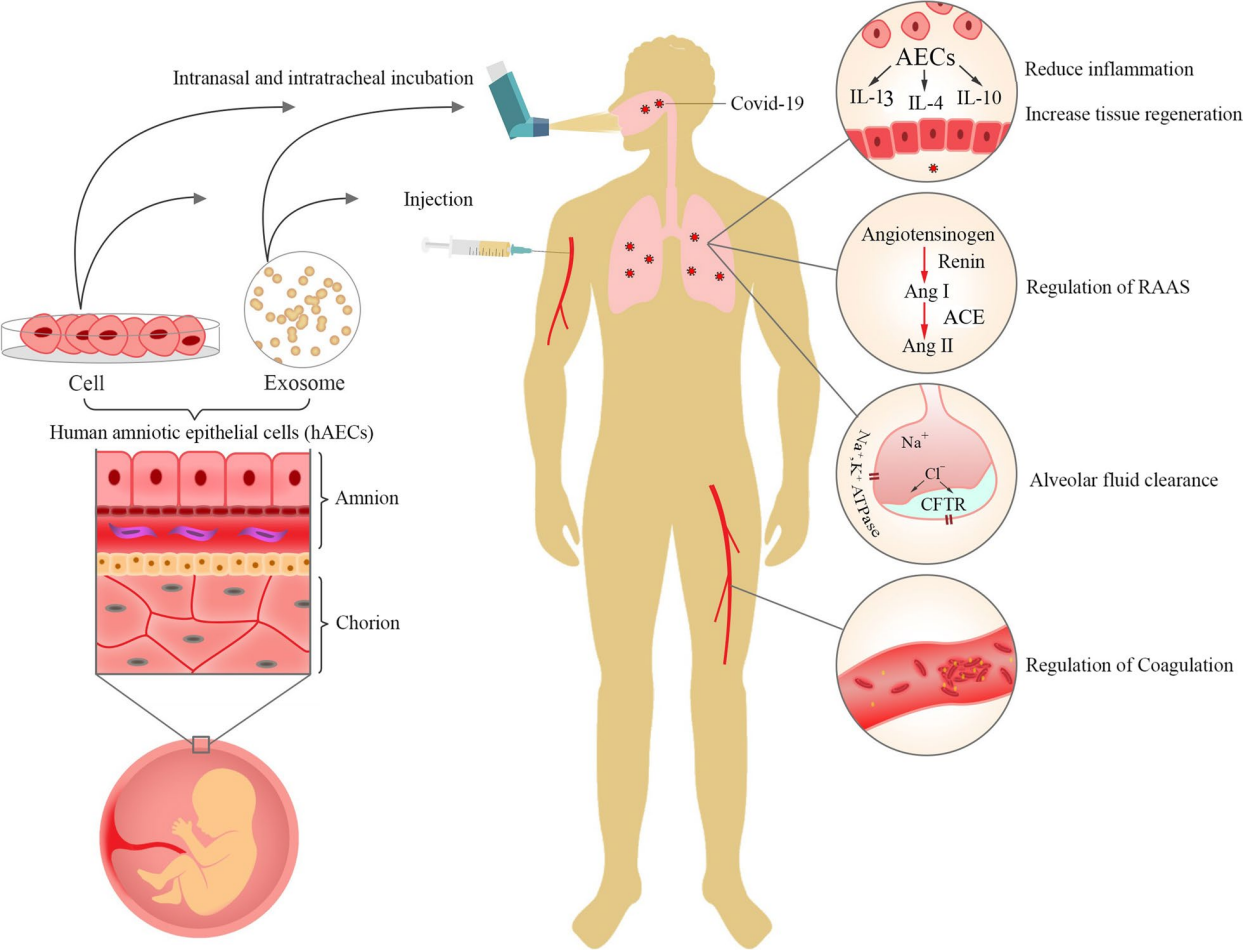

## Introduction

Coronavirus disease 2019 (COVID-19), caused by severe acute respiratory syndrome coronavirus 2 (SARS-CoV-2), has reached a new morbidity peak worldwide. New variants of SARS-CoV-2, including B.1.1.7 from the UK, 501Y.V2 and Omicron (B.1.1.529) from South Africa, and B.1.617 from India have made the spread and complication of COVID-19 more worrying [1–4]. So far, COVID-19 was symptom-free or showed flu-like syndrome in many patients and a minority of infected cases demonstrated severe pneumonia and acute respiratory distress syndrome (ARDS), the most severe complication of COVID-19 [5, 6]. However, studies have shown that new variants can enhance disease severity and increase the virulence of the virus [7, 8]. Despite the unclear pathophysiology of COVID-19, studies attribute the most complications to the uncontrolled immune system reaction. Previously, the term “cytokine storm” was used to describe maladapted induction of immune response in COVID-19. However, considering the lower amounts of some inflammatory mediators and dynamic alteration in the concentration of many cytokines, some researchers proposed alternative descriptions such as complex viral sepsis with immune deviation to describe the immune phenomenon in COVID-19 [9]. These suggestions mean that the exact pathophysiology and the possible future complications are still under question.

In order to reduce the severe complications and mortality of COVID-19, management of COVID-19 patients mainly consists of supportive care such as invasive mechanical ventilation and extracorporeal membrane oxygenation (ECMO), and there is still no proven curative drug, especially for susceptible patients [10]. Despite introducing various COVID-19 vaccines such as Pfizer-BioNTech, Johnson & Johnson’s Janssen, Sinopharm, Oxford–AstraZeneca, and Sputnik, the lower vaccine effectiveness has been reported for some variants, including β variant (B.1.351) and Omicron (B.1.1.529) [4, 11, 12]. Besides, many reports and hypotheses are released about the long-term consequences of COVID-19, including renal, cardiovascular, hepatic, and respiratory complications [13–16]. Since we are still at the early stages of the COVID-19 pandemic, vaccines’ effect on these complications has not been evaluated yet.

Considering the current problems in COVID-19 management, there is still a need to develop practical therapeutic approaches that could reduce the early and late complications and mortality of COVID-19.

Human amniotic epithelial cells (hAECs) and their exosomes have been widely studied for various therapeutic effects on immune-related and degenerative diseases. These cells and exosomes display considerable health-giving effects via immunomodulating properties on both immune cells and cytokine production. hAECs modulate immune cells such as lymphocytes, neutrophils, natural killer cells, and macrophages as primary sources of cytokine production during COVID-19 pathogenesis [17–19]. Additionally, hAECs release some types of angiotensin (Ang), such as Ang-(1-7), which confront destroying effects of SARS-CoV-2 on the renin–angiotensin–aldosterone system (RAAS), including lung fibrosis and pulmonary hypertension [20, 21]. As a severe complication of COVID-19, ARDS could be eliminated by hAECs-derived surfactants, which reduce air–liquid surface tension in damaged alveoli and improve patients' respiratory quality [22, 23]. Furthermore, studies have reported dysregulated coagulation such as deep vein thrombosis (DVT) and pulmonary thromboembolism (PTE) in COVID-19 [24, 25]. hAECs could control thrombotic events by releasing anti-thrombotic molecules such as perlecan, hyaluronic acid, and serpin F1 [26]. Besides, hAECs have shown promising regenerative potential in lung-related diseases, which could be used as a therapeutic approach in chronic complications of COVID-19 such as lung fibrosis [27, 28].

Within this context, comprehensive literature searches were performed using Scopus, PubMed, Medline, Embase, Clinicaltrials.gov, in order to identify publications written in English up to January 2022. Besides, the used keywords were stem cell, amnion, amniotic epithelial cell, exosome, regenerative medicine, cell therapy, immunomodulation, inflammation, lung fibrosis, renin–angiotensin–aldosterone system, alveolar fluid, coagulation, coagulopathy, COVID-19, SARS-CoV-2, drug delivery, gene modification and immune system focusing on the biology of hAECs and the possible therapeutic role of hAECs in COVID-19. We merged founded records and removed the duplicates using EndNote X9 (Thomson Reuters, New York, NY, USA). In order to increase the accuracy, two reviewers screened the publications by title, abstract and full texts to exclude unrelated records independently. Finally, three expert reviewers extracted data from all qualified studies.

Herein, we discuss the mechanisms involved in therapeutic features of human amniotic epithelial cells (hAECs) and their exosomes, the role of preconditioning, and gene modification of these cells to be used as a drug delivery system, and recent clinical trials in COVID-19.

## Human amniotic epithelial cells

Human amniotic membrane contains two primary cell types, including human amniotic epithelial cells (hAECs), human amniotic mesenchymal stromal cells (hAMSCs), which can be isolated through appropriate methods [29]. Single-layer hAECs is located on a compact basement membrane adjacent to amniotic fluid. Beneath this structure is a stroma containing amniotic mesenchymal cells (AMCs) that secrete basement membrane contents such as collagen, laminin, and fibronectin. Amniotic epithelium originates prior to gastrulation, suggesting that some cell populations from this area could show pluripotency [30]. hAECs are differentiated from amnioblasts which originated directly from the epiblastic layer during the early gestational period. Unlike adult epithelial cell populations, it has been shown that they retain their stemness properties [31]. These multipotent cells can differentiate into mesodermal, ectodermal, and endodermal lineage cells [32]. It has been observed that hAECs express stem cell pluripotency markers such as octamer-binding-protein-4 (OCT-4), fibroblast growth factor (FGF)-4, tumor rejection antigens (TRA) 1-60, TRA-1-81, stage-specific embryonic antigen (SSEA)-3, and SSEA-4, as well as high rates of Homeobox protein NANOG that helps embryonic stem cells maintain their pluripotency [33]. However, it has been shown that they are a heterogeneous population consisting of mature, multipotent, pluripotent, and progenitor cells that express different grades of pluripotency markers [34]. One of the main reasons for this heterogeneity is that the hAECs lose some of their stemness during the gestational period [35]. For instance, it has been shown that during 16–17 weeks of gestation, a higher percentage of hAECs express pluripotency markers such as NANOG, OCT4, and SOX-2 compared to the epithelial cells isolated from the term placenta [36]. Despite their differentiation and proliferation potency, hAECs do not present the properties of teratogenicity that have been observed in the other type of stem cells [37, 38]. It seems that the lack of telomerase activity and expression of tumor rejection antigens (TRA) in hAECs can lead to tumor suppression [38].

There are many studies on the isolation of hAECs after elective Cesarean section. The isolation methods are mainly based on peeling of chorion from amnion and separation of hAECs from the underlying basement membranes via enzymatic digestion by enzymatic solutions such as trypsin/EDTA and dispase II, and some detergents [39, 40]. Although some other cell types can be presented in the final isolated cells, studies have shown that the final isolated cell population mainly consists of hAECs under standard isolation [40, 41]. The identification of epithelial cells during the culture is based on morphologic characteristics and expression of epithelial and pluripotency markers. The epithelial cells can be flat, columnar, and cuboidal, while the mesenchymal cells are spindle-shaped [34]. Also, they express epithelial markers such as CK 1-8 and E-Cadherin [34]. After the isolation, density separation techniques can be used for identifying and proliferating hAECs with higher expression of stem cell markers such as SSEA-4 [42]. Other Stem cell markers such as TRA-1-60, TRA-1-81, and SSEA-3 have also been used for identifying hAECs with higher grades of stemness [43–45].

After the isolation, an average of 100 million cells of amniotic epithelium could be obtained from each amnion (about 100 million cells). After six passages, this cell number could even be increased to 10–60 billion by adding epidermal growth factor (EGF), which acts as a mitogen and enhances the proliferation of hAECs [46]. Besides, isolating the same cell numbers takes about four hours for hAECs, while it takes approximately 4–6 weeks for AMCs [47]. Based on the current studies, the appropriate cell number for intravenous (IV) administration of stem cells in treating COVID-19 patients is about one million cells per kilogram of weight; thus, the cells isolated from each placenta without any further passage could be enough for nearly 100 infected patients in one administration dose [48, 49].

They also affect host tissue cells by paracrine effect and consequently promote regeneration. hAECs have the immune privilege potency due to low expression of human leukocyte antigens such as HLA-A, HLA-B, HLA-C, HLA-DR while presenting immunomodulatory HLA-G [50, 51]. This feature reduces the chance of immune rejection in the human body. The other property which makes hAECs a candidate for cell therapy is their anti-inflammation feature. They increase the expression of interleukin (IL)10, a potent anti-inflammatory cytokine, and matrix metalloproteinase (MMP) 9, which has an anti-fibrotic effect. They also decrease PDGF secretion from macrophages M2, suppressing the immune response [52]. hAECs can reduce inflammation and the following fibrosis by secretion of TGF-β3 and suppression of TGF-β1. The TGF-β group includes three subtypes, TGF-β1 and TGF-β2, which have proinflammatory and fibrosis effects, and TGF-β3 with reverse potential. TGF-β3 mainly has anti-inflammatory and anti-fibrotic roles by antagonizing TGF-β1 [50]. Accumulating pieces of evidence have suggested that hAECs can provide a desirable microenvironment for tissue recovery by releasing bioactive cytokines and extracellular vesicles [53, 54]. It has been shown that hAECs can release anti-fibrotic, proangiogenic, and anti-inflammatory factors such as vascular endothelial growth factor (VEGF), angiogenin (AGN), and insulin-like growth factor (IGF) [55].

hAECs secreted extracellular vesicles (EVs) contain various bioactive molecules that can affect the microenvironment for inducing different therapeutic effects [56]. Utilizing exosomes instead of cells offers attractive benefits, such as calculating dosage and potency, storage capability, more stability, and an increased chance of crossing the blood–brain barrier (BBB) [57]. However, exosomes therapy has some blind spots such as lack of regenerative and proliferation capacity, as well as the inability of targeted migration to the injury site. Thus, whether using exosomes or cells are completely distinctive approaches with exclusive pros and cons which should be considered regarding pathology and injured organ situation [58]. Advantages of hAECs and their exosomes, including availability, low cost, and desirable biological features would bring them up as a proposed therapeutic approach in COVID-19. Herein, we discuss different therapeutic mechanisms of hAECs such as immunomodulation, renin–angiotensin–aldosterone system (RAAS) regulation, alveolar fluid clearance, coagulopathy elimination, and regenerative capacity in COVID-19.

## COVID-19 pathophysiology and the potential therapeutic effects of amniotic epithelial cells

### Immunomodulatory effect of hAECs in COVID-19

Although earlier reports focused on respiratory failure in the COVID-19, it has been observed that besides the lungs, SARS-CoV-2 is capable of invading various organs, including kidneys, brain, and gastrointestinal tract [59–61]. Additionally, the response of the host immune system is also a determining factor regarding the course of the disease. In general, the course of the COVID-19 in severe cases can be divided into three stepwise phases. In the first phase, known as the viral replication phase, the SARS-CoV-2 invades host cells and replicates. This invasion initiates both innate and adaptive immune system responses. However, some patients' inadequate or misdirected immune response can lead to the second phase, known as “cytokine storm,” resulting in a hyperinflammatory response and systemic inflammation [62]. This hyper-inflammation can cause ARDS, multi-organ failure, and death in the last phase. Understanding the bimolecular reactions in each phase can guide researchers into new preventive and curative actions.

After arriving at its destination, SARS-CoV-2 fuses with the host cells using Spike (S) proteins. Since previous reports suggested that angiotensin-converting enzyme (ACE)-2 was involved in the invasion of host cells by SARS-CoV and other coronaviruses, ACE-2 was one of the early culprits that have been investigated for its potential role in SARS-CoV-2 invasion [63]. Later findings indicated that ACE-2, with the help of other membrane proteins such as transmembrane serine protease 2 (TMPRSS-2), binding immunoglobulin protein (GRP78), and CD-147, an MMP inducer expressed in epithelial cells and WBC, acts as a target for the S2 domain of SARS-CoV-2 [64–67]. ACE2 is expressed on various cells, including the lung alveoli, gastrointestinal tract, brain, kidney, and liver. The co-expression pattern of ACE-2 with TMPRSS-2, GRP78, heat shock protein-70 (HSP-70), and CD-147 can decide the pattern of the symptoms and prognosis of patients [68].

After the host cells are invaded by SARS-CoV-2, the innate immune system and adaptive immune system are activated through paracrine signaling [69]. Since the complete activation of both the humoral immune system that produces neutralizing antibodies and the cellular immune system may need days to be completed, the early responses to the infections can be attributed to the innate immune system [70]. Interactions between infected cells and antigen-presenting cells (APCs) trigger innate immune system activation [71]. To activate the innate immune response, the single-strand RNA of the virus (ssRNA) interacts with pattern recognition receptors (PRRs) such as toll-like receptors (TLRs) and retinoic acid-inducible gene-I-like receptors (RLRs), which are expressed in pneumocytes, dendritic cells, monocytes, and macrophages [72, 73]. TLR-7 triggers the Janus kinase (JAK)-signal transducer and activator of transcription (STAT), activator protein 1 (AP-1), interferon regulatory factors (IRF)-3, IRF-7, and nuclear factor kappa-light-chain-enhancer of activated B cells (NF-κB) [73, 74]. The NF-κB upregulates the production of IL-1, IL-6, tumor necrosis factor (TNF)-α, and interferon (IFN)-α, which are the central cytokines involved in the hyperinflammatory response in patients with COVID-19 [75]. The production of these cytokines attracts the cells of the innate immune system as well as lymphocytes. Additionally, the innate immune system tries to eliminate infected cells by secreting defensins and other antimicrobial peptides, reactive oxygen species (ROS), and neutrophil extracellular traps (NET), as well as producing more pro-inflammatory cytokines [9, 76].

The IFN-α has a crucial role in defending the host cells against the invading virus. However, the N protein of the SARS-CoV-2 redirects the signaling pathways from IFN-α producing cascades to IFN-β producing cascades, resulting in a hyper-reactive but inadequate innate immune response [77, 78]. In COVID-19, it has been observed that the number of resident alveolar macrophages has been significantly decreased. In contrast, there has been an increase in the number of cytokine-producing pro-inflammatory-monocyte-derived macrophages [79]. The overproduction of the pro-inflammatory cytokines can result in a cytokine storm and an exaggerated immune system response that can lead to acute respiratory distress syndrome (ARDS) [62]. The response from the adaptive immune system leads to an increase in the number of CD4+ cells, CD8+ cells, and Th-17 in the invasion sites [80]. The activation of the adaptive immune system will also further upregulate the NF-κB signaling cascade, which is responsible for the secretion of IL-1, IL-6, and TNF-α [81]. Furthermore, activation of the adaptive immune system will result in the production of IL-8, monocyte chemoattractant protein (MCP), granulocyte colony-stimulating factor (G-CSF)-α, TNF-α, prostaglandin E2 (PGE2), and IL-17 [82]. This overproduction of cytokines by both the innate and adaptive immune systems can lead to T cell exhaustion. The exhausted T cells (Tex), which can be identified by the presentation of specific markers such as CD279 and CD 366, have a reduced level of functionality and cannot provide an efficient immune response against SARS-CoV-2 [83]. In this regard, the lower number of CD3+ cells, CD4+ cells, CD8+ cells, CD19+ cells, and natural killer (NK) cells was observed in severe cases of COVID-19 [80]. It means that regulatory mechanisms that inhibit the overproduction of pro-inflammatory cytokines have also been disrupted. The uncontrolled overproduction of cytokines primarily by the innate immune system will result in a cytokine storm and a systemic hyper-inflammatory/immunodeficiency state. In this phase, the local immune system response turns into systemic inflammation and multi-organ failure [84].

Amniotic epithelial cells have been successfully used in treating several conditions such as bronchopulmonary dysplasia, lung injury, the aftermath of cerebrovascular accidents, and liver fibrosis [85, 86]. As mentioned earlier, the COVID-19 course includes a viral phase, a cytokine storm phase, and a multi-organ hyper-inflammation phase. The immunomodulatory features, anti-inflammatory and anti-fibrotic, and antimicrobial characteristics of hAECs can be beneficial in the viral phase, cytokine storm, hyper-inflammation phase, as well as repairing the degenerative damages in the lung tissue.

hAECs can regulate immune system response by altering the expression pattern of inflammatory cytokines through paracrine signaling or direct cell contact. hAEC can potentially control the overproduction of cytokines and prevent cytokine storms. TNF-α and IL-6 are two of the most contributing factors in cytokine storm and occurrence of ARDS [87, 88]. AECs release some small molecules like migration inhibitory factor (MIF), IL-10, and PGE2, which can modulate cytokine-producing inflammatory cells and prevent the cytokine storm [89]. In a study conducted by Murphy et al., hAECs suppressed inflammation and promoted remodeling in mice with lung injury. This study demonstrated that levels of inflammatory cytokines such as TNF α, TGF-β, IF-γ, IL-6 have significantly decreased after hAECs injection [27]. Furthermore, it was observed that injection of hAECs prevented infiltration of inflammatory cells. In another study with similar aims, it was observed that hAECs decreased MCP-1, TNF-α, IL-1, IL-6, and TGF-β [90]. It is also worth noting that these immunomodulatory features of hAECs depend on regular host immune function and cannot be observed in patients with impaired immune systems. The innate immune system plays an essential role in hAECs immunomodulatory effect since observations indicate that the anti-inflammatory properties of hAECs are dependent on host normal macrophage function [91].

hAECs modulate the proliferation and differentiation of both the adaptive and innate immune system cells [17]. These cells present markers such as CD59 and FasL that modulate adaptive immune system response. Moreover, the HLA-G presented by hAECs regulates the differentiation of Treg, myeloid cells, and NK cells and prevents hyperinflammatory reactions [92]. As mentioned earlier, the cytokine storm is triggered by exaggerated innate immune system response and uncontrolled stimulation of the adaptive immune system. hAECs can prevent the differentiation of dendritic stem cells through contact inhibition and prevent unnecessary triggering of both the innate and adaptive immune systems at the beginning [93]. They also have shown promising results in reducing neutrophil infiltration and reducing oxidative bursts. Furthermore, it has been observed that in lung injuries, hAECs alter the polarization of macrophages and shift pro-inflammatory M1 macrophages into M2 macrophage, which can secrete anti-inflammatory cytokines like IL-10 through paracrine signaling [94]. Observations also indicated that hAECs could decrease the production of cytokines produced by CD4+ cells, Th1 cells, and Th17 cells while increasing cytokines produced by Th2 through IL-5 signaling cascade [95]. The shift toward the IL-5 pathway modulates hyper-inflammatory responses and improves the humoral immune system response. hAECs could help the maturation of FOXP3-expressing Treg cells, which can modulate inflammatory responses [96]. In another study, it was observed that hAECs could reduce the amount of pro-inflammatory cytokines such as IL-17 and caused an increase in the number of Treg and CD4+ T cells after an ischemic cerebrovascular accident (CVA) [97]. An increased proportion of Th2 cells in the peripheral lymphoid organs and within the central nervous system (CNS) were also observed in this study. It is also worth mentioning that hAECs do not suppress the immune system but prevent hyper-inflammation. An interesting observation in this study was prevention of pneumonia which is a common occurrence in the aftermath of CVA due to temporary immunocompromisation [98].

The hAECs secrete antimicrobial peptides (AMPs) such as human β-defensin (HBD) 1, HBD2, HBD3, secretory leukocyte protease inhibitor, and elafin [99]. We previously showed that inflammatory cytokines such as IL-1β significantly upregulates the secretion of these antimicrobial peptides [100]. HBDs, which naturally are present in the mucosa, can trigger the activation of the innate and adaptive immune system and play an essential role in the early immune response to prevent the spread of the virus [101]. Furthermore, HBD3 has shown excellent antibacterial properties that can prevent nosocomial secondary bacterial infections in COVID-19 [102].

In recent years, exosomes have been the subject of many studies. It has been suggested that exosomes extracted from hAECs have anti-inflammatory properties. It has been shown that AEC-derived exosomes were enriched for factors that participate in immunomodulation, such as P38, PI3K‐Akt, and proteins involved in Fc‐gamma receptor-dependent phagocytosis pathways. The hAECs-derived exosomes increased macrophage's phagocytic activity and altered their polarization state mainly through the PI3K‐Akt pathway. Exosomes could also reduce neutrophil myeloperoxidase and suppress CD3/CD28 activated T cell proliferation [103].

### Effects of hAECs on renin–angiotensin–aldosterone system in COVID-19

The RAAS supervisory system is extended in different body organs that consist of several components, including angiotensinogen, various angiotensin (Ang) types, ACE, and AT1 receptors. ACE-1 converts Ang I to Ang II, which occurs extensively in the lung. Ang II can induce inflammation, profibrotic pathways, thrombosis, and vasoconstriction via the angiotensin II type 1 receptor (AT1R). Another arm of RAAS acts through the ACE-2-Ang-(1-7)-Mas receptor axis, in which the activity of ACE-2 leads to conversion of Ang II to the Ang-(1-7). This axis possesses counterregulatory actions on Ang II and results in vasodilation and reducing inflammation [63, 104]. The contributive roles of various components of RAAS in some viral infections are already established. Early studies investigated and confirmed the contribution of ACE-2 in SARS-CoV-2 invasion. SARS-CoV-2 infiltrates the host cells’ cytosol using their envelope-anchored spike (S) glycoproteins binding to the ACE-2 cellular receptors [105]. Nearly all body organs express this receptor, especially type 2 alveolar epithelial cells [106]. The interaction between the viral spike protein and ACE-2 promotes the activation of metalloproteinase 17 (ADAM-17), which results in shedding and inhibition of ACE-2 [107]. Inhibition of ACE-2 redirects the RAAS axis toward ACE 1-Ang II-AT1 receptor, which is a lung-destructive factor by inducing fibrosis, inflammation, vasoconstriction, and alveolar epithelial cells apoptosis [108].

Amniotic epithelial cells can reduce the harmful effects of ACE 1-Ang II-AT1 receptor axis on pulmonary tissues. It has been demonstrated that Ang-(1-7) is presented in amniotic epithelial cells adjacent to the implanted embryo that indicates possible luminal secretion of these peptides. Besides, increased Ang-(1-7) secretion at late gestation was observed in the uterus and placenta. The presence of Ang-(1-7) induces inhibitory effects on Ang II that results in vasodilation and lower inflammation. It is hypothesized that the higher amount of Ang-(1-7) may be attributed to other enzymes such as neprilysin and prolyendopeptidase [20]. Some studies reveals that the amounts of ACE2 and TMPRSS2 are correlated to pregnancy stages. The expression of both ACE2 and TMPRSS2 are reduced gradually during first trimester and second trimester and it is undetectable in third trimester placenta [109]. Placental absence of ACE2 and TMPRSS2 as critical gateway of SARS-CoV-2 entry protect hAECs against possible infection during COVID-19 cell therapy. This protective ability prevents exclusion of hAECs from therapeutic process and provides safe cell therapy regardless of possible viral infection.

### Lung-related alveolar fluid clearance effect of hAECs in COVID-19

Alveolar fluid clearance (AFC) impairment in COVID 19 can lead to related lung disease poor outcomes due to gas exchange disturbance and ARDS and/or coinfections. To achieve an efficient AFC, it is crucial to establish a tight alveolar epithelial barrier, as well as set a sufficient ions gradient across the alveolar epithelium in which sodium (Na^+^) plays a pivotal role. The sodium–potassium adenosine triphosphatase pump (Na/K-ATPase) actively provides the Na+ gradient. Subsequently, Na+ enters the epithelial cells through the epithelial sodium channel (ENaC), which reabsorbs water in the alveolar space. Besides, the Na/K-ATPase pump, localized at the lateral and basal sides of the epithelial cells, works as a tight junctional molecule and a regulator for the cytoskeletons network and the tight junctions’ permeability. SARS-CoV-2 can alter the expression and function of the Na/K-ATPase pump by elevating levels of inflammatory cytokines, including IL-1β, IL-6, IL-8, and TNF-α [110]. TNF-α can also alter sodium uptake and chloride secretion by reducing the expression of the ENaC mRNA and the stability of the cystic fibrosis transmembrane conductance regulator (CFTR) as a chloride channel, which can result in higher airway liquid film depth and pulmonary edema. Notably, type II alveolar cells use CFTR as the chloride channel for the active clearance of alveoli from water. In addition, TNF-α increases the permeability of the pulmonary capillary endothelial cells and alveolar epithelial cells through induction of cytoskeleton disruption [111].

Lung surfactant principally reduces the surface tension at the air–liquid interface in alveoli, prevents lung collapse, and facilitates breathing. Surfactant consists of phospholipids (80%), cholesterol (10%), and four surfactant proteins (SP), including the hydrophilic SP-A and SP-D (also named collectins), and hydrophobic SP-B and SP-C [112]. Type II alveolar cells are responsible for the secretion and recycling of surfactants. It has recently been suggested that administration of the surfactant can reduce pulmonary edema, improve blood oxygenation, regulate the uncontrolled inflammatory response, and facilitate respiratory effort in patients with critical COVID 19 pneumonia. Even though lung surfactant therapy is a standard and efficient treatment for ARDS, natural surfactants appear more beneficial than recombinant surfactants [113]. In addition, the intratracheal application of the SP-C-based surfactant has demonstrated immunoregulatory features and improved gas exchange [114]. Although the pulmonary anti-inflammatory properties of surfactants are well known, their non-pulmonary immune function is also noteworthy. For instance, the topical administration of surfactants on the skin has improved wound healing with less fibrotic composition through a significant reduction in the level of proinflammatory cytokines TNF-α and IL-6 [112]. Ujma et al. have reviewed the anti-inflammatory and antimicrobial properties of hydrophilic surfactant proteins (SP-A and SP-D) in tissues such as gastrointestinal and female reproductive tracts by reducing TNF- α and IL-1 in various sites like skin, eye, ear, nose, artery, CNS, and gastrointestinal, urinary, and female reproductive tracts, which can be possible targeted sites for SARS-CoV-2 and its co-infections [115].

The contribution of the hAECs in the secretion of pulmonary surfactant into amniotic fluid has been identified. It has been demonstrated that hAECs express all four surfactant proteins SP-A, SP-B, and SP-C, and SP-D. Moreover, the hAECs express ATP binding cassette subfamily A membrane 3 (ABCA3), a lamellar body protein requisite for pulmonary surfactant construction that also expresses in alveolar type II cells. This study also showed that dipalmitoyl phosphatidyl choline (DPPC), one of the predominant components of the surfactant phospholipids and an essential contributor to surfactant biophysical functionality, could be detected in hAECs-derived supernatant [22]. These results confirmed that hAECs could express and secrete all four types of surfactant proteins with potential biophysical performance to reduce air–liquid surface tension.

The potential ability of the hAECs for differentiation to the alveolar epithelial type II cells can also bolster the hypothesis of utilizing hAECs for improving AFC in COVID 19 patients [88]. Besides, this study shows that hAECs administration represents lung fibrosis abrogation and repair of lung injury through anti-inflammatory features, such as decreasing TNF- α, IL-1, IL-6, TGF- β, and MMP-1 2. Formerly, it has been indicated that hAECs produce the tissue inhibitor of metalloproteinase (TIMP) type 1 and specifically type 2, which prevent inflammatory effects of MMPs [116]. In this regard, hAECs can potentially interfere with the destructive effect of COVID-19 on alveolar fluid clearance. These cells can preserve the transepithelial ion movement by regulating the inflammatory-induced down-regulation of ENaC and CFTR in lung injuries and COVID-19. A study on the combination of the hAECs and surfactant at clinically-used concentrations determined that this exposure has no damaging effect on the viability, functionality, and phenotype of the hAECs. Besides, the hAECs could differentiate to alveolar type II epithelial cells in small airway epithelial growth medium regardless of treating with surfactant [117].

To conclude, the distinctive properties of hAECs in clearing the alveolar space of fluid brought the convincing idea of utilizing these cells for patients with severe COVID 19. This can also be achieved by co-administrate of the hAECs and surfactants.

### Effects of hAECs in COVID-19 hypercoagulopathies

COVID-19-associated dysregulated coagulation is one of the major complications that could be seen in COVID-19 infection. This condition could cause life-threatening exposures such as deep vein thrombosis (DVT), thromboembolism, platelet aggregation, and thrombosis. In a cohort study of 184 COVID-19 patients in the intensive care unit (ICU), the cumulative incidence of large-vessel thrombotic events was 49%, and pulmonary embolism was the main observed event in lung computed tomography (CT) [118]. The extensive expression of ACE2 receptors within endothelial cells simplifies the SARS-CoV-2 binding and entrance, causing infection, vascular injury, and endothelial dysfunction [119, 120]. Circulating cell-free (CF) nuclear materials (e.g., DNA, RNA, and histone) that are released in the infectious situation due to cellular and tissue necrosis, apoptosis, autophagy, or mitotic catastrophe is known as NETosis [121]. Neutrophil extracellular traps (NETs) are DNA structures combined with histones and antimicrobial proteins that are released during cell injuries, especially by neutrophil cytotoxic enzymes [122]. Circulating cell-free nuclear materials could be a cofactor for coagulation activation factors VII, XI, and XII-activating proteases and provoke a thrombotic response [123, 124]. NET-associated histones could cause platelet aggregation by TLRs, which exist on platelets and other cells’ surfaces [125]. Pro-inflammatory cytokines such as IL-6, IL-17A, and TNF-α are enhanced in moderate to severe condition patients. These pro-inflammatory cytokines significantly induce clot formation and platelet hyperactivation and disrupt physiological anticoagulant pathways [126]. Studies have shown that inhibition of IL-6 leads to the elimination of thrombin production. IL-1 and TNF-α show their effects by downregulation of thrombomodulin and inhibiting tissue plasminogen activator [127]. Vascular injury and disintegrated intercellular junction due to COVID-19 could expose the subendothelial matrix, including tissue factor (TF) and collagen [128]. Furthermore, in COVID-19, TF expression on macrophages and platelets is enhanced by inflammatory cytokines and tissue factor pathway inhibitor (TFPI), which inhibits the TF pathway, is impaired by inflammation. As a result, the coagulation cascade is activated, and an increase of fibrin level raises the risk of clot formation in the blood [129, 130].

Expression of IL-10 by hAECs inhibits coagulation by suppressing monocytes. Monocytes trigger coagulation cascades by activating tissue factor (TF) and stimulating thrombin production [26]. Furthermore, two types of glycosaminoglycans, including perlecan and hyaluronic acid are secreted from hAECs that inhibit thrombosis by inducing endothelial cell proliferation, which is necessary to suppress the initiation of thrombosis. Pigment-epithelium derived factor (PEDF) is another released factor from hAECs that inhibits platelet activation and aggregation through antioxidant capacity. MMP-9 secreted by amniotic cells can also prevent platelet activation by suppressing the Na+/K+ exchanger and adjusting intracellular calcium balance [26]. Platelet activation results in the adhesion of platelets to each other that leads to the release of their granule substances, such as P-selectin, which is mainly known for participating in platelet rolling during the clot formation process [131]. Research indicates that incubating platelets with hAECs can considerably reduce P-selectin levels and consequently expand prothrombin time (PT) and prevent thrombosis [132]. Furthermore, hAECs express anti-inflammatory factors like IL-4, IL-10, and IL-13 to suppress inflammation and downregulate pro-inflammatory cytokines IL-1 and TNF-α [26]. The latter pro-inflammatory factors show procoagulant properties by inhibiting three critical substances of the anticoagulation procedure, including plasminogen activator, thrombomodulin, and protein C [127]. The proposed therapeutic effects of hAECs and common mediators are shown in Table 1.

**Table 1:** Proposed therapeutic effects of hAECs and responsible mediators in COVID-19

Mediators	Mechanism	Effects	References
MIF	Immunomodulation	Prevention of cytokine storm	[27, 89]
IL-10			
PGE2			
HLA-G	Immunomodulation	Regulation of immune cells differentiation	[92]
IL-5	Immunomodulation	Modulation of hyper-inflammatory responses	[95]
		Improvement of humoral immune system	
AMPs	Immunomodulation	Triggering early immune response	[101, 102]
		Prevention of nosocomial secondary bacterial infections	
Ang-(1-7)	Regulating RAAS	Reduction of inflammation, fibrosis, thrombosis, and vasoconstriction	[20, 63, 104]
SP-A	Alveolar fluid clearance	Reduction of air–liquid surface tension	[22]
SP-B			
SP-C			
SP-D			
TIMP-1	Alveolar fluid clearance	Prevention of inflammatory effects of MMPs	[116]
TIMP-2			
IL-10	Eliminating hypercoagulopathies	Suppression of coagulation cascades by inhibiting monocytes-induced activation of TF	[26]
Perlecan	Eliminating hypercoagulopathies	Inhibition of thrombosis by inducing endothelial cell proliferation	[26]
Hyaluronic acid			
PEDF	Eliminating hypercoagulopathies	Inhibition of platelet activation and aggregation through antioxidant capacity	[26]
MMP-9	Eliminating hypercoagulopathies	Prevention of platelet activation by suppressing the Na+/K+ exchanger and adjusting intracellular calcium balance	[26]
IL-4	Eliminating hypercoagulopathies	Inhibition of plasminogen activator, thrombomodulin, and protein C	[26, 127–130]
IL-10			
IL-13			

MIF, migration inhibitory factor; IL, interleukin; PGE2, prostaglandin E2; HLA, human leukocyte antigen; AMP, antimicrobial peptide; Ang, angiotensin; SP, surfactant; TIMP, tissue inhibitor of metalloproteinase; PEDF, pigment epithelium-derived factor; MMP, matrix metalloproteinase; RAAS, renin–angiotensin system

## Tissue regenerative ability of hAECs in COVID-19

COVID-19 can affect the tissues of the body, especially in the respiratory system. It has been demonstrated that 17% of the COVID-19 patients had ARDS, among whom 65% died of the disease worsening [133]. MMPs released in the inflammation phase of COVID-19 can damage the epithelium and endothelium; thus, the tissue could progress to fibrosis. Some substances secreted by epithelial cells, such as pro‐fibrotic growth factors, chemokines, vascular inhibitors, and procoagulant mediators activate the fibroblasts and play a key role in lung fibrosis [134]. In the post-COVID-19 patients who have experienced ARDS during their disease, irreversible and progressive pulmonary fibrosis is one of the critical long-term complications [135]. Besides, the cardiovascular system might be affected by COVID-19; however, the underlying pathophysiology is unclear. Several mechanisms such as ACE2-dependent myocardial infection, myocyte apoptosis due to excessive extracellular Ca^2+^ level caused by cytokine storm, myocardial injury due to increased myocardial demand in an acute infection, arrhythmia, and abnormal blood pressure are supposed for cardiac damage [136–138]. Kidneys are another organ that COVID-19 could damage. Sepsis caused by cytokine storm and direct attack of the virus through ACE-2 and dipeptidyl peptidase-4 (DPP-4), which are expressed on tubular cells, have been known as binding seats for SARS-CoV [139]. Liver injury with elevated hepatocyte-derived enzymes and serum bilirubin has been observed in COVID-19 patients. Studies showed that 14.8–53.1% of COVID-19 patients had abnormal levels of alanine aminotransferase (ALT) and aspartate aminotransferase (AST) during the disease, with a mostly mild rise in serum bilirubin which suggested hepatocyte damage [140]. The mechanism of liver injury is not precise, but it may be due to direct virus-induced cytopathic effects and/or immunopathology induced by overshooting inflammatory responses and drug-induced injury during the treatment of the disease [141]. COVID-19 can cause acute or chronic neurological deficits that could raise the risk of neurodegenerative diseases [142]. COVID-19 also attacks the endothelium of the vessel's wall. It was described that the cytokine storm and the inflammation afterwards could detach the endothelial cells. Whatever the severity of the disease is higher, the count of detached endothelial cells increases [143]. Considering the involvement of various body organs and possible tissue damage, proposing an effective regenerative approach for reducing tissue damage-related late complications is necessary.

Regenerative medicine is a promising treatment for chronic and disabling situations [144]. Transplanted stem cells participate in tissue regeneration via two main mechanisms: first, by direct differentiation of cells toward mature and functional cells, and second, via their paracrine impacts. It seems that paracrine influences are essential in tissue regeneration compared with direct differentiation. Paracrine effects consist of various cytokines, growth factors, and differentiation-promoting agents that play critical roles in tissue repair and regeneration [145]. hAECs have many capabilities, such as stem cell-like proliferation, differentiation, and immunomodulatory features [146]. Considering tissue damages due to SARS-CoV-2 infection and dysregulated immune response, hAECs could participate in the regeneration of COVID-19 affected organs.

The differentiation potential of hAECs makes them a source for cell replacement therapy [147]. The hAECs express some lung cells specific markers such as thyroid transcription factor or Nkx 2.1, which is among the earliest lineage specification markers of the developing lung. These markers give hAECs the potential to differentiate into type II alveolar pneumocytes and produce surfactants [88, 148]. In addition to the differentiation properties, hAECs-derived exosomes have a crucial role in regeneration medicine. Exosomes of hAECs contain P38 protein, which is critical factor for tissue regeneration. These exosomes contain miR-23a and miR-203a, which inhibit Smad 2 and 3, respectively, as well miR-34a that induces lung fibroblast senescence. The mentioned miRNAs cause the suppression of TGF-β and fibroblast proliferation [103]. Besides, it has been shown that the miRNAs content of hAEC-derived exosomes participated in their regeneration ability [149]. Tan et al. showed that hAECs could decrease tissue damages by inhibiting myeloperoxidase (MPO) produced by neutrophils [103]. hAECs application also can prevent lung injury by reducing the number of neutrophils and macrophages in the lung and inhibiting the overexpression of inflammatory cytokine genes such as TNF-α, IL-1, IL-6, and IFN-gamma [150].

In hypoxic cardiac damage, hAECs secrete pro-angiogenic cytokines, including angiogenin (ANG), epidermal growth factor (EGF), monocyte chemoattractant protein (MCP)-1, and IL-6 that have angiogenic effects through several pathways, including paracrine effects and chemoattractant-mediated effects [151]. When cultured hAECs are exposed to TGF-β1, Epithelial-to-Mesenchymal Transition (EMT) can occur. EMT would enhance the cardiac regeneration capacity of hAECs by increasing their mobility and extracellular matrix-modulating capacity [152].

In kidney injury, intravenous injection of hAECs diminished the apoptosis and necrosis of peritubular capillaries and tubular cells and provoked cell proliferation. hAECs decrease the number of pro-inflammatory M1 macrophages and shift them to anti-inflammatory M2 macrophages. This shift is associated with the increased IL4 and IL13, and reduced levels of TNFα and IFNγ, which reduce the inflammation response [153]. hAECs motivate the expression of miR-101 in CD4+ T cells in acute kidney injury (AKI) patients that inhibit the activation of CD4+ T cells. As a result, IL-2, which plays a crucial role in kidney injury, is reduced in inflammation [154].

Native hAECs express hepatocyte-related genes such as alpha (1)-antitrypsin, glutamine synthetase, phosphate synthetase-I, phosphoenolpyruvate carboxykinase, cytochrome P (CYP)-450, CYP2D6, and CYP3A4. When hAECs are cultured in an in vitro environment, they express transthyretin, tyrosine aminotransferase, CYP2C9, and the key hepatocyte transcription factors, HNF-3g and C/EBP-a genes [155]. In vitro, hAECs differentiate into hepatocyte-like cells with the fetal hepatocytes features, whereas in vivo, they differentiate into adult hepatocytes [156]. It has been shown that transplanted hAECs reduced collagen deposition and diminished the pro-inflammatory and pro-fibrotic cytokines in an experimental animal model of liver fibrosis [157].

**Fig. 1:**
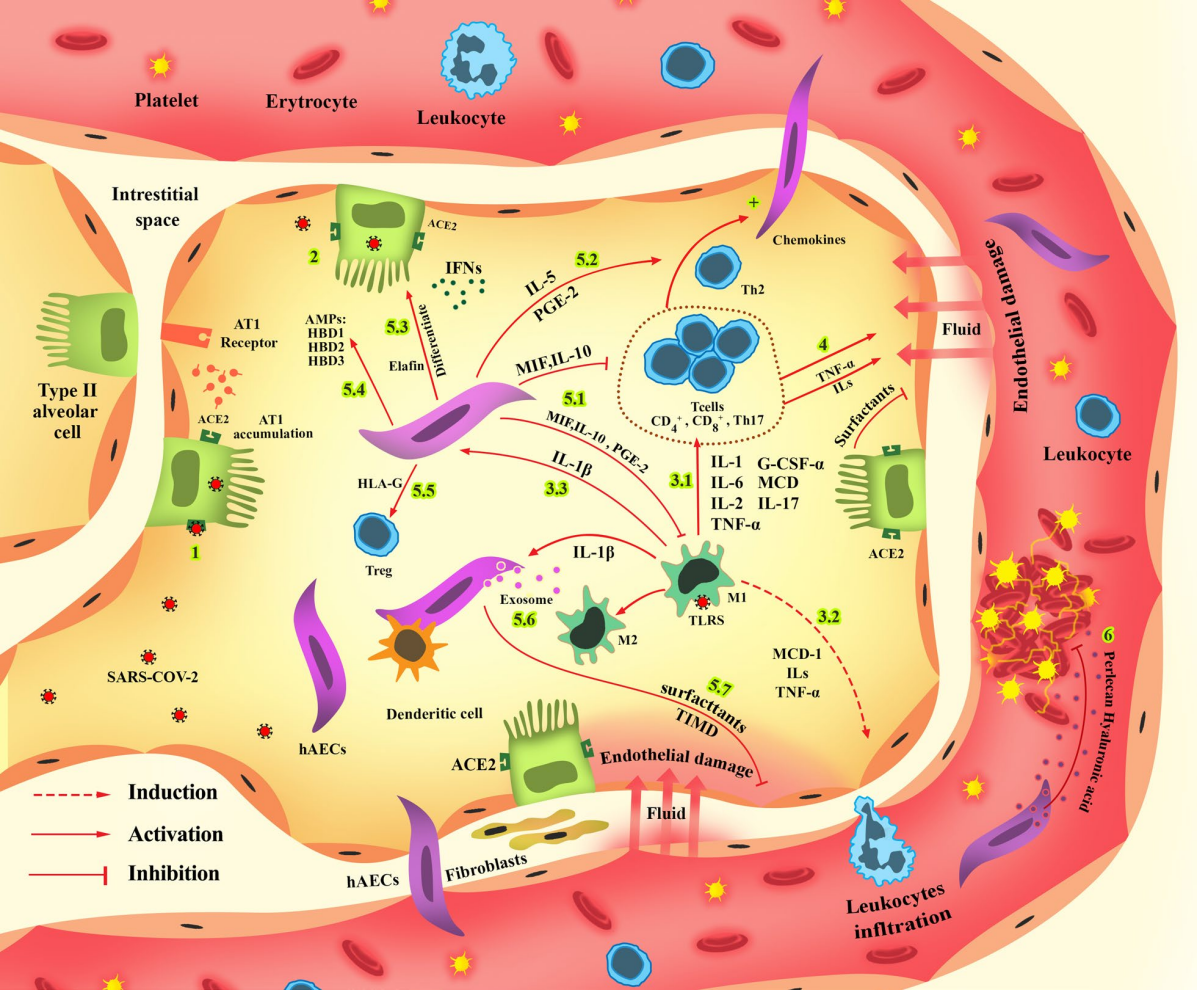
Proposed therapeutic mechanisms of hAECs in COVID-19. (1) SARS-CoV-2 enters the respiratory system and attaches to ACE2 receptors located on the basolateral membrane of type II alveolar cells. ACE2 damage would result in AT1 accumulation in alveoli that could induce vasoconstriction, inflammation, fibrosis, and apoptosis of alveolar epithelial cells. (2) Interferons, especially type I, are released from infected type II alveolar cells that defend against the invading virus. (3.1) M1 macrophages are activated in the immune response process that release IL-1, IL-6, IL-8, TNF-alpha, G-CSF-alpha, MCP, IL-17, and IL-5. These factors activate the CD4+, CD8+, and Th17 T cells. (3.2) MDC, IL-5, and TNF-alpha have chemotaxis effects that cause the migration of leukocytes to the alveolar space. (3.3) IL-1β secreted by M1 macrophages could activate the hAECs. (4) Activated CD4+, CD8+, Th17 cause inflammation and cytokine storm that damage the endothelial and epithelial cells and alveolar fluid accumulation. T cells also have chemotaxis properties that can call the hAECs to the alveolar space. (5.1) hAECs release PGE-2, IL-10, and MIF that inhibit the activation of macrophages, CD4+, CD8+, and Th17 cells and modulate cytokine-producing inflammatory cells. (5.2) hAECs induce inhibitory cytokine production in T -helper 2 through IL-5 signaling cascade. (5.3) hAECs can be differentiated into alveolar cells that regenerate damaged lung tissue. (5.4) hAECs secrete AMPs such as HBD1, HBD2, HBD3, secretory leukocyte protease inhibitor, and elafin. AMPs play an essential role in the early immune response that reduces the spread of the virus. Furthermore, they have antimicrobial properties that prevent nosocomial infection. (5.5) HLA-G presented by hAECs can regulate the differentiation of Treg cells, preventing hyperinflammatory responses. This feature also reduces the chance of immune rejection in the human body. (5.6) Activated hAECs release some exosomes which contain regenerative agents and PI3K‐Akt pathway activators that induce M1 to M2 macrophage polarization. (5.7) hAECs release surfactants and TIMP that prevent the accumulation of the fluid in the alveoli. (6) hAECs secrete two types of glycosaminoglycans, perlecan and hyaluronic acid, that inhibit thrombosis. Thus, hAECs could play an important role as an inhibitor of clot formation in coagulation dysregulation caused by COVID-19. Abbreviations: hAECs, human amnion epithelial cells; ACE, angiotensin-converting enzyme; AT1, angiotensin 1; IL, interleukin; G-CSF, granulocyte colony-stimulating factor; MCP, monocyte chemoattractant protein; MDC, macrophage-derived chemokine; PGE, prostaglandin; MIF, macrophage migration inhibitory factor; AMPs, antimicrobial peptides; HBD, human beta-defensin; HLA, human leukocyte antigen; TIMP, inhibitor of metalloproteinase

The therapeutic potential of hAECs is also seen in the treatment of neurological injuries and diseases. hAECs could differentiate into the neural cells. Moreover, they have paracrine effects by secreting cytokines, neurotrophic factors (NTFs), growth factors, and neurotransmitters that facilitate neural survival and regeneration, axonal outgrowth, and synapse reformation, thus leading to the reinnervation of lost neuronal connections and further recovery of neurological functions [158]. Considering the regenerative features of hAECs, including releasing many regenerative factors, differentiation to intended cells, and creating an appropriate reconstructive environment, they could emerge as a suitable regenerative agent in COVID-19 degenerative complications. The possible therapeutic effects of hAECs are shown in Fig. 1.

## Modification of hAECs for administration in COVID-19

### Preconditioning and gene modification of hAECs

Although cell-based therapy (CBT) has shown promising potential in treating various disorders, its efficacy needs to be promoted to reach desirable outcomes. One of the major causes of poor therapeutic performance of transplanted cells is the host's harsh environment. Hypoxia, acidosis, and excessive inflammation in COVID-19 prevent administered cells from establishing sufficient function needed for the favorable therapeutic function [159]. Preconditioning is a promising strategy to overcome the negative impacts of the harsh environment on administered stem cells. Preconditioning is applied on the cells before transplantation in order to adjust them to the host’s milieu and improve their function [160]. Preconditioning has various methods such as hypoxic preconditioning, acidic preconditioning, and pharmacologic preconditioning to enhance cell survival, anti-oxidative capacity, angiogenesis induction, migration, and immunomodulatory impacts [161–163].

In COVID-19, the body’s destructive environment causes cell death and dysfunction due to various harassments such as hypoxia and acidosis [164, 165]. As a promising stem cell modification strategy, preconditioning could be utilized. Hypoxic preconditioning is shown to improve the survival and performance of administered cells in hypoxic conditions. The transcription factor hypoxia-inducible factor-1*α* (HIF-1*α*) is upregulated during hypoxic stress. HIF-1*α* induces transcription of survival-related genes such as PI3K/Akt and ERK1/2, as well as proliferation-related genes such as Nrf-2 [166–168]. Acidic preconditioning could also improve hAECs therapeutic potential. It has been demonstrated that acidic preconditioning of hAECs improves their survival and hampers apoptosis through upregulation of PI3K/Akt and ERK1/2 pathways [169].

Oxidative stress occurs in various conditions, such as ROS accumulation during hypoxia. The excessive ROS production by neutrophils causes tissue damage, thrombosis, and red blood cell dysfunction in critically ill COVID-19 patients [170]. Preconditioning with H_2_O_2_ and hypoxia is shown to improve stem cells’ anti-oxidative capacity through upregulation of genes such as Nrf-2, heme oxygenase-1, autocrine motility factor, and hexokinase-2 [171, 172]. Enhanced antioxidative activity promotes cell survival after injection and improves their therapeutic impacts against COVID-19 [170]. It has been reported that glial cell line-derived neurotrophic factor (GDNF) preconditioning of amniotic cells improves stem cells’ resistance against hydrogen peroxide-induced cytotoxicity. Moreover, preconditioning of amniotic cells with GDNF improves their migration and engraftment to the injured tissue by upregulating mobility and engraftment-related factors, including CD44, C-X-C chemokine receptor type 4 (CXCR4), and CX3CR1 [173].

Since the immunomodulatory features of hAECs are pivotal in COVID-19 treatment, it is therapeutically beneficial to improve cells’ immunomodulatory capacities. Depending on the stem cells’ microenvironment, they can shift toward both inflammatory and anti-inflammatory phenotypes. Preconditioning with high amounts of inflammatory cytokines such as TNF-α and IL-6 increases the shift towards anti-inflammatory phenotypes [174, 175]. It has been elucidated that preconditioning of hAECs with remifentanil (an opioid analgesic) alleviated the production of IL-1β and TNF-α proinflammatory factors when exposed to an inflammation inducer lipopolysaccharides (LPS). Remifentanil preconditioning also decreases the expression of COX-2, a cytokine with proinflammatory impacts [176].

Gene modification is a cell manipulation strategy in which specific genes are upregulated or downregulated through various mechanisms such as utilizing viral vectors. It has been shown that gene modification of hAECs improves their viability, differentiation, and metabolic characteristics.

It has been reported that enhancing GDNF gene expression by gene modification improved amniotic cells’ survival under oxidative stress and inflammatory conditions and promoted their migration and engraftment into the damaged site. Besides, GDNF gene-modified amniotic cells had augmented levels of HIF-1α, transforming growth factor beta 1 (TGF-β1), and VEGF production. Reduced oxygen supply and tissue ischemia result in mitochondrial injury, mitochondrial ROS accumulation, and mitochondrial bioenergy dysfunction, leading to cell apoptosis. It has been shown that GDNF gene modification improved the anti-oxidative capacity of amniotic cells and preserved mitochondrial integrity and bioenergy. Upregulation of GDNF through gene modification also promoted cells’ anti-inflammatory capacity. Transplanted gene-modified amniotic cells into mice models of obstructive nephropathy decreased MCP-1 and TNF-α inflammatory cytokine levels robustly.

### hAECs as a drug delivery tool

In recent years, many efforts have been made to use hAECs as drug reservoirs due to their low immunogenicity, prolonged circulation time, and ability to controlled release. Drugs loaded on stem cells have higher concentrations at the target site on account of stem cell homing ability to the damaged tissues. Moreover, these drugs possess reduced side effects and higher bioavailability due to lower kidney or hepatic clearance [177–179].

In this regard, some studies tried to load antiviral agents and antibiotics on AECs. It is demonstrated that AECs treated with acyclovir and trifluridine antiviral drugs inhibited replication of viruses in cell culture, showing their potential for usage as a drug reservoir for antiviral purposes [180]. Besides, amniotic cells could be an appropriate choice for ofloxacin delivery, a quinolone antibiotic, in a sustained drug release manner [181]. Moxifloxacin is a fourth-generation fluoroquinolone used for the treatment of bacterial infections. It has been reported that amniotic cells could be a proper choice for moxifloxacin drug delivery since they can release the drug even 7 weeks after loading of drug [182]. Amniotic cells treated 30 min with netilmicin, an aminoglycoside antibiotic, inhibited Staphylococcus epidermidis infection for at least 3 days. These cells could uptake netilmicin in a dose-dependent manner in which the higher drug dose results in higher uptake by amniotic cells [183].

It seems that the idea of applying drug-loaded and modified hAECs is a hopeful and encouraging strategy as a therapeutic approach to encounter complications and progression of COVID-19.

## Routes of delivery

For successful cell-based therapy, the administered stem cells must migrate to the target tissue. Migration is the arrival of stem cells from the delivery site to the target tissue, which occurs through the circulation system in systemic administration strategies [184]. Various molecules are involved in the migration and homing process of stem cells, such as chemotactic agents, adhesion molecules, and MMPs. Chemotactic agents such as CXCR4 promote stem cell tendency to the injured area and aid the migration process, leading to a successful homing. Adhesion molecules such as ICAM-1, ICAM-3, and integrins are responsible for stem cell adhesion and incorporation into the target tissue. MMPs such as MMP-2 and MMP-9 digest the target tissue’s extracellular matrix (ECM) to facilitate stem cell embedment into the target tissue [185–187].

Different routes are available for hAECs delivery into the body, including intravenous injection, intratracheal, and intranasal routes. In addition, hAECs could be administered intraperitoneally in in vivo experimental studies [86, 88, 150, 188]. As discussed, administered stem cells migrate to the injury site with the assistance of systemic circulation, chemotactic agents, released inflammatory cytokines from the damaged tissue, and cell–cell adhesion molecules [87]. The most desired delivery route has the fewest side effects, appropriate accessibility, and the highest cell number in the injury site.

Intravenous injection (IV), as the most common and feasible systemic administration route, has an advantage for treating pulmonary disorders, which is called the “pulmonary first-pass effect”. It has been demonstrated that most IV injected stem cells are trapped into the lung, which augments the administered cells concentration in the damaged pulmonary site, which is beneficial for treating COVID-19 [189]. It has been shown that hAECs could migrate to the injured pulmonary tissue, differentiate into type I and II alveolar cells, and mitigate ventilation-induced lung injury [190]. Nevertheless, IV administered stem cells must reach the target site via systemic circulation, which diminishes their population in the target tissue compared to the local routes such as intranasal and intratracheal [191].

Intranasal and intratracheal administrations have been shown to improve stem cells’ viability and population in the target tissue. In addition, intranasal delivery is a non-invasive route which makes it an attractive choice. However, some disadvantages are associated with these routes, including the need for intubation for intratracheal delivery and a limited number of cells that could be administered through these routes [87].

It has been shown that hAECs-derived exosomes possess many therapeutic effects. Exosomes could be administered through different routes such as intravenous, intranasal, subcutaneous, and oral. The intravenous route is the most frequent route for exosome administration due to its favorable feasibility. It is shown that exosomes can escape the immune system-mediated removal and liver clearance when administered intravenously. Moreover, exosomes are trapped into the lung during intravenous administration, similar to transplanted hAECs, which could be beneficial for the treatment of pulmonary disorders. Intranasal administration is a proper and non-invasive administration route for exosome delivery into the systemic circulation, and in some studies, it has been demonstrated that intranasal exosome delivery is even more efficient compared to IV injection [190, 192–194].

## Pre-clinical and clinical application of hAECs

Considering various therapeutic potentials of hAECs, evaluating the possibility and safety of administration of these cells in animal models and humans is an essential step in developing AEC-based therapeutic approaches. Similar to drugs, Cellular and Tissue-Based Products (HCT/Ps) safety is a prior issue in clinical application. It has been shown that the most tolerated dose and the no observed adverse effect level (NOAEL) of hAECs used in a mouse model were both higher than MSCs. Besides, administering hAECs even at the most tolerated dose had no adverse effect on growth, behavior, and general histology of animal models [195]. Tumorigenicity, another concerning issue in using HCT/Ps, is the main reason for failure in clinical trials on cell-based therapy [196, 197]. As mentioned above, some features of hAECs, including restricted proliferation due to the limited telomerase function, prevent tumorigenicity of hAECs [33]. Accordingly, the use of hAECs in animal models has not shown tumor formation that ensures another pivotal aspect of safety for clinical application of hAECs [195]. In another preclinical study, Hodges et al. utilized in utero ovine model of ventilation that caused ventilation-induced lung injury (VILI) to investigate whether hAECs would relieve lung injury. It has been shown that hAECs could reduce profibrotic mediators such as TGF-β1 and pro-inflammatory cytokines like IL-8 [190]. Vosdoganes et al. administered hAECs in bleomycin-induced pulmonary inflammation and fibrosis. They observed that intraperitoneally administration of hAECs returned lung tissue density, collagen content, and α-smooth muscle actin (α-SMA) production to a normal condition. It also reduced pulmonary leukocyte infiltration and expression of TGF-β, PDGF-α, and PDGF-β in the lungs [198]. The same study revealed that intraperitoneal administration of hAECs could downregulate proinflammatory cytokines such as TNF-α, TGF-β, IFN-γ, and IL-6 and also diminish subsequent pulmonary fibrosis by lowering pulmonary collagen deposition, levels of α-SMA, and inflammatory cells infiltration [27]. In another preclinical study, intranasal administration of AEC-derived exosomes in bleomycin-induced lung fibrosis reduced lung inflammation after 1 day. Besides, the treatment on the seventh day enhanced the tissue-to-airspace ratio and considerably ameliorated lung fibrosis [103]. The pre-clinical uses of hAECs in lung injuries are summarized in Table 2.

**Table 2:** Pre-clinical use of hAEC in lung disease

No	Pathologic condition	Type of HCT/Ps	Mechanism of action	Administered dose	Route of administration	References
1	Lipopolysaccharide-induced lung injury	hAECs	Reducing lung inflammatory mediators	1.8 × 10^8^ (intratracheal)	Intratracheal	[86]
				0.9 × 10^8^ (intravenous)	Intravenous	
2	Lung fibrosis	hAECs	Surfactant production	1 × 10^6^	Intravenous	[88]
			Differentiation to pneumocytes			
			Immunomodulation			
3	Bleomycin-induced lung injury	hAECs	Reducing lung inflammatory mediators	4 × 10^6^	Intraperitoneal	[27]
			Reducing leukocyte infiltration			
			Preventing collagen deposition			
4	Bleomycin-induced lung injury	hAECs	Reducing macrophage infiltration	4 × 10^6^	Intraperitoneal	[94]
			Switching M1 to M2 macrophages			
5	Idiopathic pulmonary Fibrosis	hAEC-derived exosomes	Promoting proliferation of bronchioalveolar stem cell Increasing macrophage phagocytosis Reducing neutrophil myeloperoxidase Suppressing T cell proliferation	10 μg	Intranasal	[103]
6	Ventilation-induced lung injury	hAECs	Differentiated into type I and II alveolar cells	120 × 10^6^ (total	Intratracheal	[190]
			Reducing fibrosis	l dose)	Intravenous	
7	Bleomycin-induced lung injury	hAECs	Reducing lung inflammation	4 × 10^6^	Intraperitoneal	[198]
			Reducing fibroblast activation			
8	Ventilation-induced lung injury	hAECs	Reducing T cell infiltration	0.9 × 10^8^ (both routes)	Intratracheal	[199]
					Intravenous	
9	Bronchopulmonary dysplasia	hAECs	Reducing lung inflammatory mediators	0.1 × 10^6^	Intratracheal	[200]
			Reducing leukocyte infiltration		Intravenous	
10	Ventilation-induced lung injury	hAECs	Regenerating lung tissue	0.3 × 10^8^	Intravenous	[201]

When the efficacy of using hAECs in pathologic conditions is approved in pre-clinical studies, it is critical to guarantee the production and preservation of HCT/Ps under clinical-grade standards. In order to achieve acceptable standards for manufacturing procedures and controlling production quality of HCT/Ps, Food and Drug Administration (FDA), Health Canada, and European Medicines Agency (EMA) have considered particular guidelines called “Good Manufacturing Practice (GMP)” [202]. Pandemic situations and limitations such as time restriction, unavailability of appropriate equipment, and financial issues may influence fulfilling GMP standards. Viral contamination is a fundamental problem in preparing HCT/Ps during the COVID-19 pandemic. Contamination can originate from different sources, including operation room, placenta, biological raw materials, manufacturing environment, and administration clinics. However, testing clinicians, related personnel, and the final products by utilizing validated tests for SARS-CoV-2 along with strictly adopting guidelines of GMP would reduce the chance of contamination [203].

As the first clinical use, hAECs have been administered in premature infants with bronchopulmonary dysplasia (BPD), a chronic lung disease with bronchi damage and alveolar dysplasia. Allogeneic hAECs were administered by intravenous infusion to six premature babies with BPD, and they were followed for 2 years. By utilizing an inline filter and lowering the concentration and the rate of infused hAECs, no adverse effects, including local site reaction, anaphylaxis, infection, features of rejection, or tumor formation was observed [204].

Some clinical studies have been launched to assess the safety and efficacy of hAECs in pathologic conditions. Up to January 2022, our search by the keywords “Amniotic epithelial cell” revealed 24 registered clinical trials of hAECs application in clinicaltrial.gov, of which seven are active and recruiting patients, and one has completed its trial. These clinical trials evaluate the efficacy of hAECs in various pathologic diseases such as bronchial fistula (NCT02959333), spastic cerebral palsy (NCT03107975), primary ovarian insufficiency (NCT03207412), infertility (NCT02912104), nonunion fracture (NCT03031509), corneal epithelial dystrophy (NCT00344708), acute-graft-versus-host disease (NCT03764228), Asherman's syndrome (NCT03223454), and Parkinson's disease (NCT04414813). To the best of our knowledge, there is only one clinical trial on utilizing hAECs for treating complications of COVID-19 in which Sandora et al. tried to regenerate cardiomyocytes damages due to myocardial infarction (MI) as an essential complication of COVID-19. They utilized a heart patch consisting of an amnion bilayer seeded with hAECs and autologous cardiomyocytes of patients who underwent coronary artery bypass graft (CABG) surgery. Autologous cardiomyocytes were isolated from waste tissues of the patient's heart after CABG surgery. They also used HLA-DR negative hAECs to reduce the risk of rejection. After the intervention, the condition of patients was evaluated by electrocardiography (ECG), echocardiography, blood test, and radiology (technetium-99m). However, no result of this study was published up to January 2022 (NCT04728906). Considering the proposed benefits of hAECs in COVID-19, including immunomodulation, alveolar fluid clearance, controlling RAAS, and regulating coagulation, along with the reported advantages of hAECs transplantation in lung injury models, these cells can be a remarkable resource for ameliorating the early and late complications of COVID-19.

## Discussion and future directions

Human amniotic epithelial cells and their exosomes can prevent or reduce hyper-inflammatory responses, tissue fibrosis, accumulation of alveolar fluid in ARDS, and severe thrombotic events. Also, hAECs can regulate transepithelial ion transportation by reversing hyper-inflammation on ENaC and CFTR in the lungs. The most prominent feature that makes hAECs a promising cell source for treating severe forms of COVID-19 is their immunomodulatory effects, which can be attributed to the regulation of NF-κB cascade and secretion of immunomodulatory cytokines such as IL-10. Furthermore, the secretion of all four natural surfactant proteins already used for treating ARDS prioritize the hAECS from other cell sources proposed to treat COVID-19. Moreover, the regeneration ability of hAECs makes them an appropriate choice for eliminating organ fibrosis as a late complication of COVID-19. Unfortunately, there are only a few reports on using hAECs in COVID-19; thus, further investigations must be conducted on the possible mechanisms, safety, and therapeutic outcomes of hAEC therapy in COVID-19. Besides, hAECs produce antiviral and antimicrobial peptides which are parts of innate immune system [99]. We believe that these hAECs-produced peptides could participate in defending the human body against SARS-CoV-2. However, further experiments need to be done to clarify the AMPs' effects on this virus. Additionally, modification strategies such as preconditioning, gene modification, and drug loading have been shown to extensively improve hAECs capacity for tissue regeneration, immunomodulation, and antiviral combatting. These strategies ameliorate hAECs survival, migratory ability, paracrine impacts, AMP production, and anti-oxidative capacity. Modification strategies are promising tools to enhance the success chance of cell therapy [159]. Nevertheless, there are only a few reports on using hAECs in COVID-19; thus, further analysis and reconsideration in the expectations of surmounting their hurdles should be performed in future studies.

## Conclusion

Human amniotic epithelial cells and their secretome would induce beneficial therapeutic effects in COVID-19 through modulating immune system reactions and controlling coagulopathies. They can also prevent lung tissue fibrosis, clear alveolar fluid, and participate in tissues regeneration in ARDS clinical and preclinical models. Modification methods such as preconditioning and gene modification can enhance the therapeutic effects of hAECs. However, there are only a few reports of administrating hAECs and their exosomes in treating COVID-19; thus, further studies are required regarding the possible therapeutic mechanisms and efficacy of hAECs in severe COVID-19 cases.

## Data Availability

Not applicable.
